# Vaccination With *Leptospira interrogans* PF07598 Gene Family-Encoded Virulence Modifying Proteins Protects Mice From Severe Leptospirosis and Reduces Bacterial Load in the Liver and Kidney

**DOI:** 10.3389/fcimb.2022.926994

**Published:** 2022-06-28

**Authors:** Reetika Chaurasia, Aryeh Salovey, Xiaojia Guo, Gary Desir, Joseph M. Vinetz

**Affiliations:** ^1^Section of Infectious Diseases, Department of Internal Medicine, Yale University School of Medicine, New Haven, CT, United States; ^2^Section of Nephrology, Department of Internal Medicine, Yale University School of Medicine, New Haven, CT, United States

**Keywords:** leptospirosis, AB toxins, immunity, vaccine, host – bacteria interaction

## Abstract

The molecular and cellular pathogenesis of leptospirosis remains poorly understood. Based on comparative bacterial genomics data, we recently identified the hypothetical PF07598 gene family as encoding secreted exotoxins (VM proteins) that mediate cytotoxicity *in vitro*. To address whether VM proteins mediate *in vivo* leptospirosis pathogenesis, we tested the hypothesis that VM protein immunization of mice would protect against lethal challenge infection and reduce bacterial load in key target organs. C3H/HeJ mice were immunized with recombinant *E. coli-*produced, endotoxin-free, leptospiral VM proteins (derived from *L. interrogans* serovar Lai) in combination with the human-compatible adjuvant, glucopyranoside lipid A/squalene oil-in-water. Mice receiving full length recombinant VM proteins were protected from lethal challenge infection by *L. interrogans* serovar Canicola and had a 3-4 log_10_ reduction in bacterial load in the liver and kidney. These experiments show that immunization with recombinant VM proteins prevents leptospirosis clinical pathogenesis and leads to markedly reduced key target organ infection in this animal model. These data support the role of leptospiral VM proteins as virulence factors and suggest the possibility that a VM protein-based, serovar-independent, pan-leptospirosis vaccine may be feasible.

## Introduction

The discoveries of the etiological agent of leptospirosis and the rodent reservoir of transmission were first reported in 1915 and 1917, respectively, and even then, was recognized as a disease of global importance (Inada et al., 1916; Noguchi, 1917). The most recent estimate of the global burden of leptospirosis conservatively estimates that this disease annually affects more than one million people, causing at least 60,000 deaths annually. Despite effective antibiotic treatment, leptospirosis still has a 5-20% case fatality rate (Costa et al., 2015; Torgerson et al., 2015; Rudd et al., 2020). There is no safe and effective vaccine generally approved for humans (Bharti et al., 2003; Brown et al., 2003; Balakrishnan and Roy, 2014; Bouvet et al., 2020; Martin et al., 2014). The lack of a human vaccine is due to several factors including reactogenicity of current bacterin-based vaccines, current concepts regarding limitations of serovar coverage, and most importantly, a lack of knowledge regarding specific vaccine antigens targeting conserved pathogenetic mechanisms (Palaniappan et al., 2007; Dellagostin et al., 2017; Bashiru and Bahaman, 2018; Felix et al., 2020).

Previous work with live (Fish and Kingscote, 1973), bacterin (Stringfellow et al., 1983; Bolin et al., 1991; Adler, 2015), lipopolysaccharide (Jost et al., 1986; Jost et al., 1989; Midwinter et al., 1990; Wang et al., 2007), recombinant proteins vaccines (Haake et al., 1999; Branger et al., 2001; Faisal et al., 2009; Grassmann et al., 2012; Llanos Salinas et al., 2020), recombinant subunit vaccine [LigA_7-13_, LigB_0-7_ (Evangelista et al., 2017), LigB_131-645_ (Conrad et al., 2017), and LigA DNA (Faisal et al., 2008)] in animals has not led to definitive vaccine candidates. Most recently an attenuated live *Leptospira* vaccine and *fcpA^-^* mutant confer cross-protective immunity against heterologous leptospiral serovars affecting humans (Murray et al., 2018; Wunder et al., 2021).

We have recently identified a family of conserved paralogous proteins encoded by the PF07598 gene family that encode virulence factors potentially involved in the pathogenesis of leptospirosis. PF07598-encoded proteins, denominated virulence modifying (VM) proteins are found only in Group 1 pathogenic *Leptospira* (Lehmann et al., 2013; Fouts et al., 2016), are secreted exotoxins with *bona fide* N-terminal ricin B domains and a C-terminal toxin domain; and their expression is variably upregulated *in vivo* in a hamster model of acute infection (Lehmann et al., 2013; Lehmann et al., 2014). After cell-surface binding, mCherry-labeled VM protein LA3490 localizes to the nucleus and mediates cytotoxicity because of potent DNase activity (Chaurasia et al., 2022).

Given these observations, we tested the hypothesis that the *Leptospira interrogans* VM proteins (encoded by the PF07598 gene family) mediate severe disease in an animal model. Targeted gene knockout is not feasible to interrupt the 12+ *L. interrogans* PF07598 paralogs to evaluate the role of individual VM proteins in mediating leptospirosis pathogenesis. Previous transposon mutagenesis experiments in which individual VM family members were disrupted did not yield informative phenotypes of disease-induction or tissue colonization (Murray et al., 2009; Marcsisin et al., 2013).

Therefore, we took an immunological approach to determine whether leptospiral VM proteins might be protective vaccine antigens in preventing severe leptospirosis in a susceptible murine model (Viriyakosol et al., 2006). We immunized mice with VM proteins identified in the *L. interrogans* serovar Lai genome: a ricin B domain (RBL-1, called t3490 here) from one VM protein (LA3490), or combinations of full length recombinant VM proteins [LA3490 (UniProt ID: Q8F0K3), LA0620 (Q8F8D7), LA1402 (Q8F6A7), LA1400 (Q8F6A9), and LA0591 (a VM protein with a similar C-terminal domain but lacking ricin B domains at the amino terminus, (Q8F8G6)], chosen according to their variable upregulation in the blood, liver, and kidney *in vivo* (Lehmann et al., 2013). After lethal challenge infection with a different serovar of *L. interrogans*, Canicola, we determined the effect of heterologous VM protein vaccination on the protection of mice from severe clinical disease.

## Results

### Conservation of PF07598 Protein Family and Their Orthologs in Pathogenic *Leptospira*

The PF07598-encoded VM paralogous protein family has an expanded repertoire within *L. interrogans*, with at least 12 distinct paralogs in serovars Lai, Copenhageni, and Canicola. Orthologs have >90% amino acid amino acid identity (**Table 1**). Most VM proteins are comprised of ~640 amino acids with an AB domain architecture comprised of two tandemly arrayed β-trefoil, N-terminal ricin B-like lectin domains, and a C-terminal toxin domain that has DNase activity (**Supplementary Figure 1**). *L. interrogans* serovars also encode a single unique ortholog that lacks a N-terminal ricin B-like domain (typified by LA0591, of ~313 aa) but which contains a signal sequence (**Table 1** and **Supplementary Figure 1**).

**Table 1:** Orthologs and percentage amino acid similarity of PF07598 gene family members in Group I pathogenic *Leptospira*.

*L. interrogans* serogroup Icterohaemorrhagiae serovar Lai strain 56601			*L. interrogans* serovar Copenha geni str. Fiocruz L1 -130			*L. interrogans* serovar		% Amino acid similarity		
						Canicola				
Gene Locus	Uniprot ID	Amino Acid	Gene Locus	Uniprot ID	Amino Acid	Uniprot ID	Amino Acid	Lai/Copenhageni	Lai/Canicola	Copenhageni/ Canicola
LA3388	Q8F0V3	631	LIC10778	Q72U83	631	A0A1R0JPK7	631	99.2	95.0	95.0
LA0835	Q8F7V7	631	LIC12791	Q72NP1	631	A0A1R0JQR7	631	99.5	92.3	91.8
LA0591	Q8F8G6	313	LIC12985	Q72N53	313	A0A1R0JVU7	313	99	97.9	99.0
LA0589	Q8F8G8	632	LIC12986	Q72N52	632	A0A1R0JW11	632	99.7	93.8	93.8
LA1402	Q8F6A7	641	LIC12339	Q72PX8	663	A0A1R0JPH9	641	100	92.3	92.3
LA1400	Q8F6A9	573	LIC12340	Q72PX7	627	A0A1R0JPC8	672	96.5	92.3	94.5
LA3271	Q8FI66	636	LIC10870	Q72TZ4	636	A0A1R0JLC7	636	98.7	95.6	95.6
LA0934	Q8F7LO	638	LIC12715	Q72NW3	638	A0A1R0JN38	638	99.8	94.5	94.6
LA0769	Q8F820	602	LIC12844	Q72NJ0	639	A0A1R0JKX7	635	98.5	96.7	97.9
LA2628	Q8F2Y3	638	LIC11358	Q72SM1	638	A0A1R0JPS3	638	99.8	99.5	99.7
Not Available			LIC10639	Q72UL8	640	A0A1R0JSB6	638	NA	NA	98.7
LA0620	Q8F8D7	637	LIC12963	Q72N74	637	A0A1R0JW34	637	99.7	97.6	97.6
LA3490	Q8FOK3	639	LIC10695	Q72UG2	639	A0A1R0JS78	634	99.7	93.6	93.6

NA, not available.

### Immunization With Full Length VM Proteins Prevented Severe Leptospirosis in Mice

**Figure 1:**
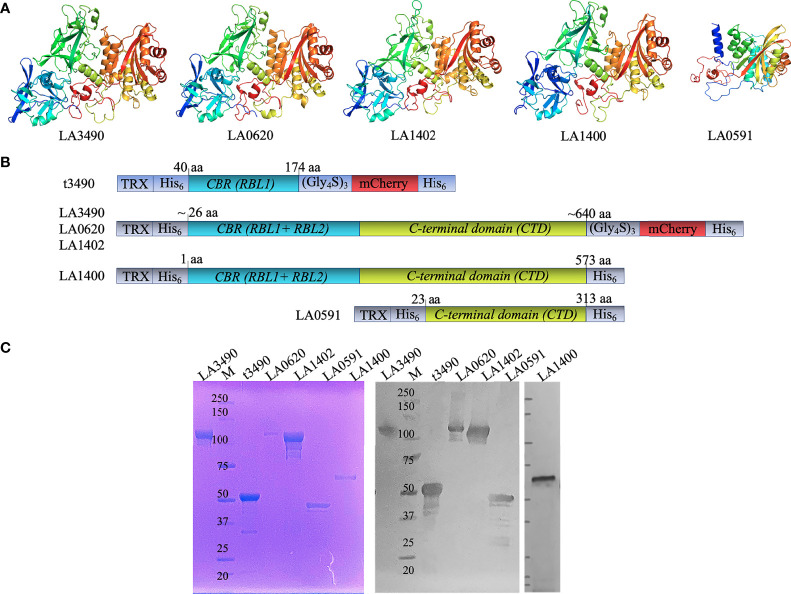
DeepMind AlphaFold algorithm derived structure, strategy for cloning, purification, and antigenicity of recombinant His-tagged VM proteins. **(A)** Artificial intelligence-based high-resolution structural modeling of (LA3490, LA0620, LA1402, LA1400 and LA0591) using AlphaFold algorithm [Callaway, E. (2020)]. **(B)** Schematic diagram depicting the organization of the recombinant mCherry (mC) fusion VM proteins used in the current study; t3490, amino acid positions 40 aa -147 aa (minus signal sequence); LA3490 (19 aa – 639 aa), LA0620 (32 aa – 637 aa), LA1402 (28 aa - 641 aa), LA1400 (1 aa - 573 aa), and LA0591 (23 aa – 313 aa). The clones were designed without signal sequences. LA1400 naturally lack signal sequence. Recombinant fusions include a glycine-serine (Gly_4_S)_3_ linker (for flexibility), N-and C-terminal His_6_ tag (purification), and N-terminal thioredoxin. **(C)** AKTA purified soluble His-tagged VM proteins (LA3490, t3490, LA0620, LA1402, LA0591, and LA1400) were analyzed by 4 ± 12% SDS-PAGE followed by Coomassie staining. A replicate gel was run for immunoblot analysis. The proteins were transferred to a nitrocellulose membrane and the blot was probed with mouse anti-His monoclonal-ALP conjugate (1:2,000 dilution; Santa Cruz Biotechnology, USA). M represents molecular weight marker.

Full length recombinant VM proteins LA3490, LA0620, LA1402, LA1400, and LA0591 (following *L. interrogans* serovar Lai nomenclature) were expressed in *E. coli* as N-terminal fusions with thioredoxin (TRX)-His_6_ affinity tags to facilitate solubility and affinity purification, and C-terminal fusions with mCherry-His_6_ to facilitate affinity purification and fluorescence microscopy visualization of the protein, respectively (**Figure 1A, B**). The homogeneity of recombinant VM proteins was verified by SDS-PAGE and Western immunoblot (**Figure 1C**).

**Figure 2:**
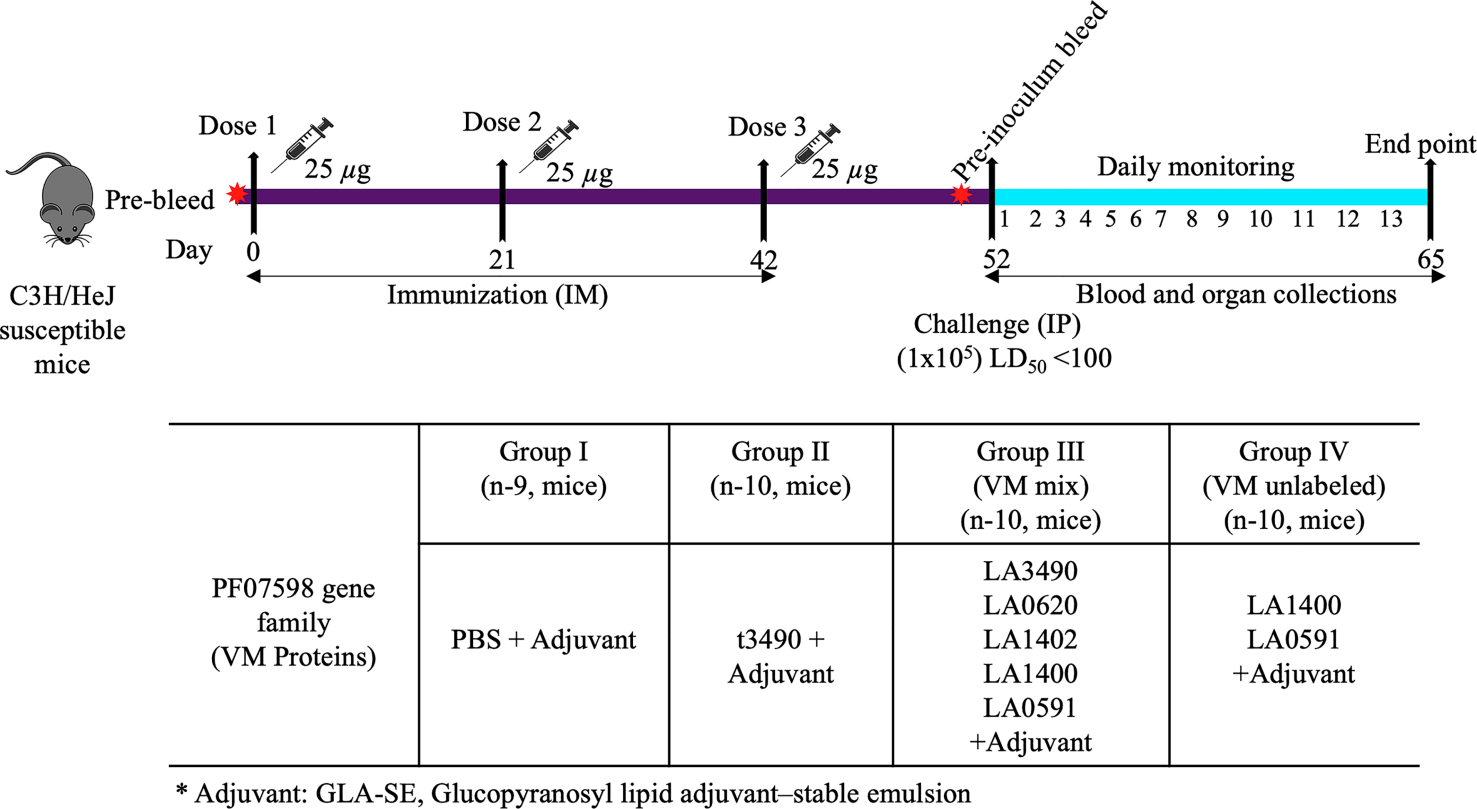
Mouse immunization schedule and sample collection. C3H/HeJ mice were immunized with 25 *μ*g of total antigen along with adjuvant (5 *µ*g GLA–squalene–oil-in-water emulsion) on days 0, 21 and 42 respectively by intramuscular route. They were pre-bled prior to each immunization and prior to challenge infection and blood was obtained on day of necropsy. Control mice were immunized with PBS buffer plus adjuvant. Following immunization on day 52, mice were infected with live *L. interrogans* serovar Canicola (*~*1x10^5^ leptospires, LD_50_ <100) by the intraperitoneal route. Blood and organs were collected after subsequent infection.* represent Adjuvant: GLA-SE, Glucopyranosyl lipid adjuvant–stable emulsion.

Mice were injected intramuscularly with recombinant proteins or PBS control mixed with glucopyranosyl lipid A/squalene oil-in-water (GLA-SE) adjuvant (schematically depicted in **Figure 2**). This adjuvant was chosen for the present experiments because it is compatible for human use, hence useful to test in animal models towards eventual vaccine development for humans. The GLA component (a synthetic, non-toxic moiety with six acyl chains on a disaccharide backbone and a single phosphate group (Pantel et al., 2012) would not be expected to have a TLR4 agonist immunostimulatory effect in C3H/HeJ mice, which are genetically hyporesponsive to lipid A due to a mutation in the gene encoding a functional Toll-like receptor (TLR4) (Beutler and Poltorak, 2000).

The primary outcome of this immunization study was whether mice developed severe manifestations of leptospirosis after lethal challenge infection (10^5^ organisms of a low passage (P3) with *L. interrogans* serovar Canicola strain LOCaS46 strain, which has a median lethal dose LD_50_ <100 (Llanos Salinas et al., 2020). Mice were euthanized and considered having arrived at a severe disease endpoint if they developed severe manifestations after a challenge infection as defined by weight loss of >15% from the beginning of the experiment or if they were unable to groom, eat, drink, or developed severe lassitude/hunching. The secondary outcomes were 1) quantitative bacterial load in the liver and kidney as measured by quantitative real time PCR, and 2) antibody responses measured by ELISA and Western immunoblots.

**Figure 3:**
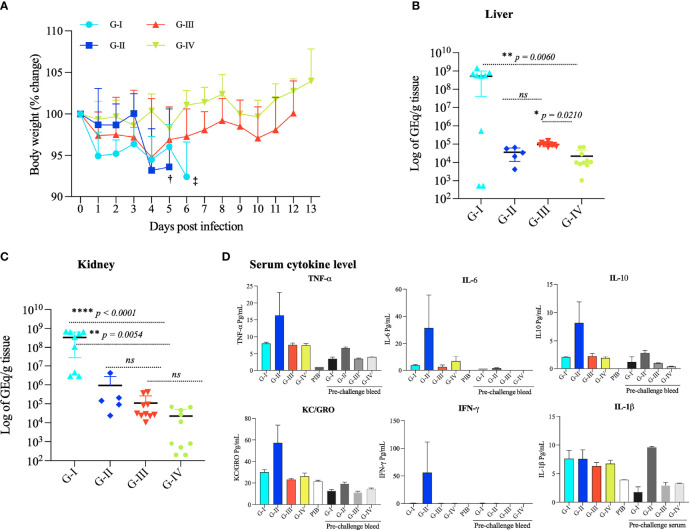
Body weight change, bacterial load, and pro-inflammatory cytokine response of mice challenged with *L. interrogans* serovar Canicola. **(A)** Mouse body weight (% change) was recorded from 0 day to 13 days upon infection; concurrent assessment of clinical status (grooming, eating, drinking, energy level) was also observed. G-I and G-II mice were sacrificed at 6^th^ and 5^th^ day (‡ and †,). Statistical analysis was performed to determine statistical significance in body weight between the PBS control and vaccinated groups using two-tailed unpaired, Mann-Whitney T-Test. *p* values: VM mix vs PBS, p - 0.0152 *: VM unlabeled vs PBS, p – 0.0005*: VM unlabeled vs VM mix, *p < 0.0001* ****: t3490 vs PBS, *p - 0.3869, ns.* Error bars indicate the standard error. Total genomic DNA was extracted from the kidney **(B)** and liver **(C)** and analyzed by qPCR performed in duplicates with *lipL32* primers and SYBER Green probes to quantify leptospiral tissue load. Statistical analysis was performed using the Kruskal–Wallis test and Dunn’s multiple comparisons test. *p < 0.0001* was considered significant. **(D)** Pro-inflammatory cytokine response in pooled serum samples from each group: G-I (PBS control), G-II (t3490), G-III (mix of 5 VM proteins), and G-IV mice (mix of 2 VM proteins) pre-challenge and post-challenge were used to measure the levels of TNF-α, IL-6, IL10, KC/GRO, IFN-γ and IL-1β by V-PLEX Proinflammatory Panel 1 Mouse Kit (Meso Scale Discovery, MD, USA), an immunoassay based on electrochemiluminescence. PIB denotes pre-immunized bleed. *p < 0.05, **p < 0.001, ****p < 0.0001.

No mouse developed severe disease after the immunization protocol. Mouse groups receiving PBS (G-I) plus adjuvant or the ricin B-domain RBL1 [t3490, (G-II)] plus adjuvant showed a modest decrease body weight after challenge infection but had to be euthanized on days 6 and 5, respectively, because of severe illness manifested by lethargy and inability to feed/drink. Vaccination with Full length VM proteins, either a mix of 5 (G-III) or a mix of 2 (G-IV), prevented all observable clinical illness (**Figure 3A**). This observation indicates that protection from severe leptospirosis required full length VM proteins.

### Immunization With rVM Proteins Significantly Reduced Bacterial Load in the Liver and Kidney

The leptospiral load of *Leptospira* in the liver and kidney in the four experimental groups was quantified by qPCR. After challenge infection, the three groups immunized with recombinant proteins plus adjuvant (G-II, G-III and G-III) had ~10^3^-10^4^ -fold fewer genome equivalents (Geq) per gram of tissue in the liver and kidney (Kruskal-Wallis test, ANOVA result: liver *p < 0.0001*, kidney *p = 0.0003*) compared to the PBS control group (G-I) (**Figures 3B, C**). Dunn’s multiple comparisons statistical test with control group PBS (G-I), VM mix (G-III) *p = 0.0054*, and VM unlabeled protein (G-IV) *p < 0.0001* confirmed this statistically significant difference.

### Immunization With t3490 Led to Severe Disease Caused by Pro-Inflammatory Cytokines Despite Significantly Reducing Bacterial Load in the Liver and Kidney

To determine whether immunization with the first highly conserved ricin B-like domain (RBL1) would confer protection from lethal challenge and as a control for the Full length VM protein LA3490, *E. coli*-produced recombinant RBL1 domain (truncated 3490, t3490) was produced and purified using identical procedures as for full length LA3490, and used for the immunization study. Surprisingly, mice (G-2) immunized with t3490 developed accelerated clinical disease after challenge infection, yet had decreased bacterial load in the liver and kidney (**Figures 3B, C**). Disease enhancement in G-2 was associated with high levels of TNF-α, IFN-γ, IL-6, IL-10, and the chemokine KC/GRO compared to the PBS and Full length protein recipient groups (**Figure 3D**).

### Antibody Profile and Cross-Reactivity of Mice Response to PF07598 (VM) Proteins Pre – and Post– Challenge

**Figure 4:**
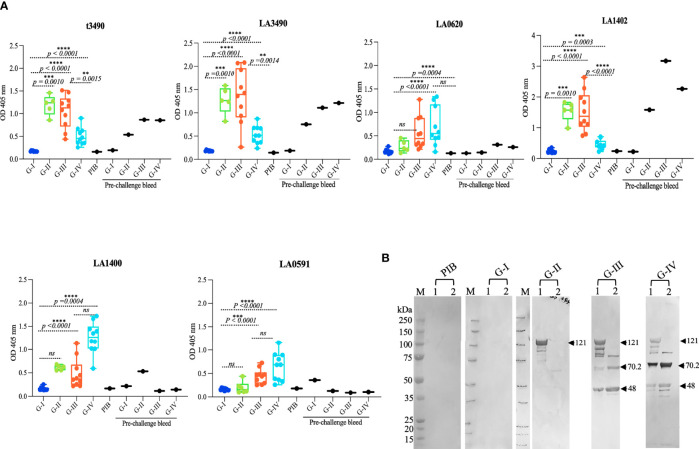
IgG responses to recombinant VM protein immunization. **(A)** Antibody titers were measured in each study group pre-and post-challenge against individual VM proteins in triplicate using ELISA. Each data line represents the average IgG response of each animal (n-10). Box and whiskers plots represent the antibody titers against t3490, LA3490, LA0620, LA1402, LA1400, and LA0591, respectively. The four-study groups include G-I: PBS, G-II: t3490, G-III: VM mix and G-IV: VM unlabeled. The box boundaries indicate the median and interquartile ranges and the whiskers denote the maximum and minimum values. Statistical analysis was performed by t-test and the non-parametric, unpaired, two tailed Mann Whitney test *p < 0.0001* values were considered significant. **(B)** Aliquot from immunized recombinant purified VM proteins were run in 4 ± 12% SDS-PAGE then transferred to nitrocellulose membrane for Western blot analysis. The membrane was probed probed with pooled sera (1:100 dilution) collected post-challenge. PIB denotes pre-immunized bleed, served as control. VM proteins were recognized by sera from G-II, G-III, and G-IV. Lane 1 shows VM mix protein (LA3490, LA0620, LA1402, LA1400 and LA0591) and Lane 2 shows VM unlabeled proteins (LA1400 and LA0591). Arrows shows expected size of VM proteins. M represents molecular weight markers. **p < 0.05, ***p < 0.001, ****p < 0.0001.

To determine whether mice immunized with VM proteins developed an IgG antibody response, sera from pre-and post-immunized mice were collected and antibody profiles were examined by ELISA using all 6 antigens used in the study (**Figure 4A**). Control group (G-I) and pre-immunized sera did not show detectable IgG antibody against any of the VM antigens. The antibody response against t3490 antigens was observed in sera from t3490 immunized mice (G-II) and cross-reactivity was seen with LA3490 (*p =* 0.0010) and LA1402 (*p =* 0.0010) antigens.

Sera from the VM mix-immunized mice (G-III) reacted with all VM antigens tested [t3490, LA3490, LA0620, LA1402, LA1400, and LA0591, (*p <* 0.0001)]; highest titers were seen against LA3490 and LA1402 antigens. The antibody responses against each antigen in the VM mix antigen group [t3490 (*p = 0.0015*), LA3490 *(p <* 0.0001*)*, LA0620 *(p = 0.*0004*)*, LA1402 *(p < 0*.0001*)*, LA1400 (*p =* 0.0003), LA0591 *(p <* 0.0001*)*] were observed with sera from VM unlabeled group (G-IV) and highest titer was detected with LA1400, LA0591, and LA0620 antigens. Antibody responses against t3490, LA3490, and LA1402 antigens were also observed in post-immunized, pre-challenge mice. Pre-challenge antibody titers to LA1400, LA0591, and LA0620 were lower than post-infection titers after challenge with live *L. interrogans *serovar Canicola. Further experimental investigation of the direct effect of VM proteins and their immune profiling *in vivo* is warranted. Despite having >90% amino acid similarity, each VM proteins showed unique reactivity with pre-and post-challenge sera and may well have different *in vivo* function. The differences in the reactivity of VM proteins indicates differences in immunogenicity and because of high level of amino acid similarity, they cross-react with pre-and post-challenge sera. Generation of VM protein-specific monoclonal antibodies and identification of protective epitopes would help to distinguish the roles of, and mechanisms by, which different VM proteins contribute to leptospirosis pathogenesis.

Cross-reactivity was confirmed by Western immunoblot analysis, probing recombinant VM proteins immobilized on nitrocellulose membrane with pooled sera from immunized animals (**Figure 4B**). The pre-bleed sera and PBS control (G-1) group did not show reactivity with a cocktail of VM mix and VM unlabeled recombinant antigens (5 and 2 proteins, respectively). Sera from t3490 immunized mice showed significant antibody titers against t3490 antigens (not shown) and cross-reacted with Full length VM proteins but faintly with LA1400, and no reactivity with LA0591, which lacks N-terminal, ricin B-domain, and suggests that t3490 only cross reacts with the epitope shared at N-terminal region of VM proteins. Sera from the VM mix group (G-III) cross-reacted with all five antigens and the reactivity pattern was consistent with each. The reactivity of LA1400 with sera from the VM unlabeled group (G-IV) was highest among all the VM proteins and in the same cocktail lot of VM antigens immunized to G-III and G-IV mice. The finding that high titer antibodies against the LA1400 antigen (as determined by both ELISA and Western blot) were induced in the VM unlabeled group sera (G-IV) suggests that LA1400 elicits the strongest humoral immune response in mice compared to other VM proteins and may be responsible mediating protective immunity. These data do provide strong confidence, however, that one or more of these VM proteins mediate the pathogenesis in this animal model. Future optimization of which VM proteins should be used for vaccination based on these observations is supported by these data.

### VM Protein Expression in *In Vitro* and *In Vivo* and Cross-Reaction Among Pathogenic Serovars

**Figure 5:**
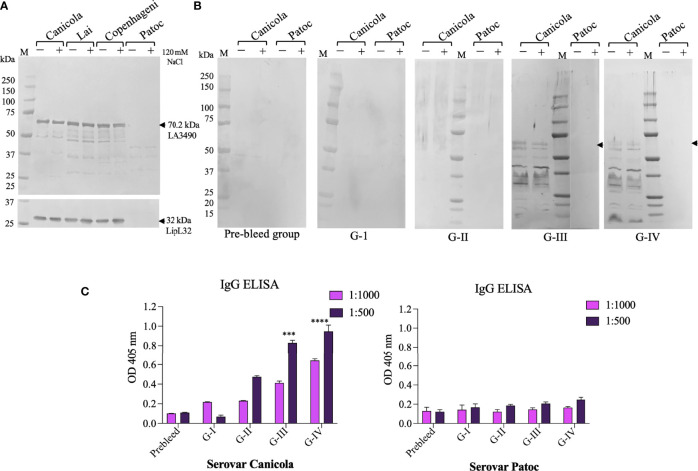
*In vitro* and *in vivo* recognition of VM proteins in *Leptospira* cell free lysate by sera from immunized mouse groups. Pathogenic *L. interrogans* serovars Canicola, Lai, Copenhagni, and non-pathogenic *L. biflexa* serovar Patoc were grown in conditional EMJH medium which was induced with 120 mM NaCl for 4 h in log phase and unconditional EMJH medium and cells were harvested. Cell-free lysates were analyzed by 4-12% SDS-PAGE, then transferred to a nitrocellulose membrane for Western blot analysis. **(A)** The membrane was probed with polyclonal LA3490 antibodies (1:2,000 dilution) and LipL32 monoclonal antibody (1:10,000) which was served as loading control. **(B)** The other set of membrane were probed with pooled sera (1:100 dilution) collected before immunization (pre-bleed) and after challenge Group I (PBS+ adjuvant), Group II (t3490), Group III (VM Mix) and Group IV (VM Unlabeled. **(A, B)** *Leptospira* grown in EMJH medium without addition of NaCl, represented by minus (–), and *Leptospira* grown in EMJH medium to log phase, at which time 120 nM NaCl was added, represented by plus (+). Arrows indicate the expression of 70.29 kDa native VM proteins. **(C)** Anti-*Leptospira* immunoglobulins generated against with serovars Canicola after experimental infection of C3H/HeJ suspectable mice. Whole cell IgG ELISA were performed with pre-bleed and sera from immunized mice post-challenge. Serovar Patoc served as negative control. ***p < 0.001, ****p < 0.0001.

Protein extracts from *L. interrogans* serovar Lai, Canicola, Copenhageni, and non-pathogenic strain *L. biflexa* serovar Patoc induced with and without 120 mM NaCl were probed on Western blots using polyclonal anti-LA3490 antibodies. Native VM protein expression was seen at the expected size of ~70 kDa molecular weight by pathogenic serovars Lai, Canicola, and Copenhageni but not with serovar Patoc (a negative control given the absence of PF07598 gene family members in this saprophytic species) (**Figure 5A**).

To determine whether immunization with a limited set of VM proteins leads to broadly cross-reactive anti-VM protein antibodies, *in vivo* VM protein expression and cross-reactive serovar immune profiles was inferred by Western immunoblot analysis using cell-free protein extracts from *Leptospira interrogans* serovar Canicola and the non-infectious saprophyte *L. biflexa* serovar Patoc. Antibodies from the immunized groups showed antibodies against serovar Canicola recognized the predicted size of VM proteins (~70 kDa), suggesting *in vivo* VM protein expression during challenge infection. This antibody reactivity also reacted with post-challenge G-III and G-IV sera. However, reactivity was not seen with the negative control, serovar Patoc cell-free lysate (**Figure 5B**). We observed lower molecular weight reactive proteins detected with sera from G-III and G-IV, suggesting the possibility that VM proteins undergo proteolytic processing (**Figure 5B**). Further study is warranted to determine whether these low molecular weight proteins play a role in leptospiral pathogenesis.

We quantified IgG antibody profiles against homologous and heterologous VM proteins. Cell-free protein extracts from *L. interrogans* serovar Canicola and the non-pathogenic strain *L. biflexa* serovar Patoc were used as solid phase antigen adsorbed to ELISA plates. ELISA confirmed reactivity of serovar Canicola with sera from the VM mix (G-III) and VM unlabeled (G-IV) post-challenge mouse groups. *L. interrogans* serovar *Lai*-encoded VM proteins that were used to immunize the G-III and G-IV groups cross-reacted with lysates of serovar Canicola. Notably, orthologs of VM protein are highly conserved in *L. interrogans* serovars and consistent with this observed cross reactivity. Non-pathogenic serovar Patoc did not cross-react with sera from control group and immunized mouse group mice either pre- or post-challenge (**Figure 5C**).

## Discussion

Mechanisms by which pathogenic *Leptospira* cause severe disease has remained elusive ever since the initial description of the etiology of leptospirosis (Inada et al., 1915; Noguchi, 1917). Although historically, the nomenclature of *Leptospira* has been confusing, recent genomic and molecular approaches have clarified the relationships among species and serovars. Of high importance is the discovery that the PF07598 gene family is present only in pathogenic, Group 1, *Leptospira* and expanded in the most pathogenic species, *L. interrogans*, as well as in *L. kirschneri* and *L. noguchii.* Severe human disease is primarily attributed to infection by serovars belonging to *L. interrogans*; such data are limited because of insufficient isolates obtained from cases of severe leptospirosis that enable definitive identification of infecting *Leptospira*. Because current gene knockout approaches to *Leptospira* remain limited, especially as applied to multi-gene families, we used an immunological approach to demonstrate whether the leptospiral PF07598 gene family-encoded VM proteins might be virulence factors contributing to severe leptospirosis disease manifestations in a mouse model. The data presented here support the hypothesis that VM proteins have central importance as virulence factors in the pathogenesis of severe leptospirosis.

Vaccination of C3H/HeJ mice with as few as two *L. interrogans* serovar Lai VM proteins (G-IV, LA1400 and LA0591) but as many as five (G-III, LA1400, LA0591, LA3490, LA0620, and LA1402) protected mice from any clinical manifestations of disease and led to ~3-4 log_10_ reduction in bacterial load in the liver and kidney, two key organs in pathogenesis of leptospirosis and transmission of *Leptospira*, respectively. Previous data indicate that all PF07598 gene family members are variably upregulated in the hamster model of acute, severe leptospirosis (Lehmann et al., 2013). We chose the specific VM protein antigens for groups G-III and G-IV based on previously data describing those with the highest and lowest expression *in vivo* (Lehmann et al., 2013). The present findings suggest that VM protein vaccination with a minimum complement of cross-reactive VM proteins might confer protective immunity but, as of yet, we do not know whether LA1400 and LA0591 are both needed as immunogens. Curiously, we found that post-immunization/pre-challenge sera from G-IV heterologously cross-reacted with highest titers against with LA1402 and LA3490 despite low titers against their homologous proteins. LA1400, an ancestral VM protein in Group 1 pathogenic *Leptospira* belonging to cluster A (Fouts et al., 2016), has 2 N-terminal, tandemly repeated ricin B-like lectin domains (RBLs), and a C-terminal toxin domain (CTD). LA0591 has a CTD but lacks RBLs. These domains might *a priori* be expected to cross react most strongly with homologous VM proteins but the experimental data indicate that heterologous cross-reactivity was instead found to be strongest. Further experiments are in progress to determine further whether immunization with either of these proteins alone, concatenated, or isolated subdomains of various VM proteins, or even one more full length VM proteins such as LA1402 and LA3490, might confer pan-leptospiral immunity. A likely scenario is that the general cross-reactivity to VM proteins induced by vaccination with LA1400 and LA0591 mediates protection against lethal challenge infection and tissue colonization. This possibility is suggested by bioinformatic analysis indicating that VM proteins are highly conserved at the amino acid level within *L. interrogans* and is supported experimentally (**Supplementary Figure 1**) (Chaurasia et al., 2022).

While immunization of leptospirosis disease-susceptible C3H/HeJ mice (Viriyakosol et al., 2006) with Full length leptospiral VM proteins protected against severe disease, vaccination with an isolated RBL, t3490, and a recombinant protein containing only the N-terminal ricin B domain (G-II), led to disease enhancement while simultaneously reducing the bacterial load in the liver and kidney. Multiplex cytokine analysis on serum showed that Group II mice had unique elevations in pro-inflammatory cytokine markers (IL-ß, IL-6, IL-10, IFN-γ, TNF-α and KC/GRO (Wolpe et al., 1989) (neutrophil chemoattractant related to IL-8 in rodents) which suggest that this cytokine storm might lead to mice death (**Figure 3D**). The mechanism by which RBD-domain-induced immune enhancement leads to severe disease is unclear. We speculate that one potential mechanism by which t3490 immunization led to disease enhancement could be the induction of antibodies to the N-terminus RBLs of *Leptospira*-secreted VM proteins that, *in vivo*, carry the Full length protein to the pro-inflammatory pathway in Fc receptor-containing cells but this hypothesis requires experimental testing. Nonetheless, cross-reactive antibody generated against RBD in G-II immunized mice did not protect against severe disease. These observations suggest that the N-terminal RBD alone should not be used for a VM protein-based leptospirosis vaccine studies. Further work to study RBD-mediated immune enhancement is needed.

In the present study, ELISA and Western blot analysis of post-vaccination sera on using recombinant VM proteins and osmolarity-induced *in vitro* cultivated *L. interrogans* serovar Lai indicates that vaccination results in both homologous and heterologous VM protein recognition associated with protective immunity. These experimental results confirm bioinformatic predictions of cross-reactivity of polyclonal antisera for VM proteins within the genus *L. interrogans*. Further work to confirm protection against challenge infection in rodent models by other *L. interrogans* serovars is essential. Cross-species protection experiments after VM protein vaccination against challenge with virulent isolates of closely related *L. kirschneri* and *L. noguchii*, the other group 1 highly pathogenic *Leptospira* species (Fouts et al., 2012; Lehmann et al., 2013) are planned. Cross-species protection experiments after homologous, or heterologous VM protein vaccination followed by challenge infection with virulent isolates of other group 1 pathogens such as *L. borgpetersenii*, which have few PF07598 paralogs in their genomes, will contribute to determining which VM proteins might be appropriate for further development into a pan-leptospirosis vaccine.

A serovar-independent pan-leptospirosis vaccine that confers protection against leptospirosis is a major priority in the leptospirosis field (Ko et al., 2009; Wunder et al., 2021). Various inactivated whole bacterial cell-based vaccines (bacterins) are serovar-specific and limited to animal use, where this legacy technology remains incompletely effective. Subunit vaccines, and more recently a spontaneously-arising attenuation mutant of *L. interrogans* serovar Copenhageni (Wunder et al., 2021) have been proposed amidst the search for pan-leptospirosis vaccine candidates (Govindan et al 2021.; Haake et al., 1999; Coutinho et al., 2011; Conrad et al., 2017; Techawiwattanaboon et al., 2019; Haake and Matsunaga, 2020; Teixeira et al., 2020; Phoka et al., 2021; Rodrigues de Oliveira et al., 2021). Bacterins are limited from wider use because of adverse effects and suboptimal efficacy, including the lack of durable protective and sterilizing immunity (Levett, 2001; Techawiwattanaboon et al., 2019; Felix et al., 2020; Zaugg and Ottiger, 2021).

The present study has several limitations. First, the clinical endpoints were visual and for ethical reasons did not use death as an endpoint. Second, the current experiments did not include histopathological analysis, nor did we attempt to isolate viable *Leptospira* from the different vaccine groups. Third, while challenge infection used a leptospiral serovar (*L. interrogans* serovar Canicola serogroup Canicola) from a different serogroup that the one from which the VM protein sequences were derived (*L. interrogans* serovar Lai, serogroup Icterohaemorrhagiae (Masuzawa et al., 1988), challenge infections using other serovars/species were not performed. This gap limits generalizability at this time but will be addressed in pending experiments. Fourth, vaccine dosing regimens—indeed the minimal set of protective VM protein antigens—have yet to be optimized. Finally, challenge infections were performed using the intraperitoneal route which is not the natural route of infection (Coutinho et al., 2014). While protective immunity in the present experiments is presumed to be mediated by antibody against *Leptospira*-secreted VM proteins, further experiments, including passive immunization and mapping of protective epitopes requiring monoclonal antibodies or putative epitope-specific polyclonal sera are needed. Nonetheless, the present report provides a strong basis for such experiments given the protective immunity induced by vaccination with a subset of *L. interrogans* VM proteins against lethal challenge infection. The immunization strategy to induce anti-VM protein antibodies validates the VM protein role in mediating leptospirosis pathogenesis.

## Methods

### Bacterial Cultures

*Leptospira interrogans* serovar Canicola strain LOCaS46 were grown at 30°C in liquid Ellinghausen-McCullough-Johnson-Harris (EMJH, BD Biosciences, USA) (Ellinghausen and McCullough, 1965). *Leptospira* were grown under conditions mimicking the *in vivo* host environment known to induce virulence gene expression *in vitro* (Matsunaga et al., 2005). Briefly, mid-logarithmic cultures in unmodified EMJH medium were harvested by centrifugation at 18,514 *g*. Pelleted cells were washed twice with 1X phosphate buffered saline, resuspended in liquid EMJH medium supplemented with 120 mM NaCl, and then incubated at 37°C for 4 h (Sigma Aldrich, USA). The LD_50_ of LOCaS46 strain has a median lethal dose LD_50_ <100 (Llanos Salinas et al., 2020).

Chemically competent *E. coli* strain DH5α (New England Biolabs, USA) was used for gene cloning, and strain SHuffle^®^T7 competent *E. coli* cells (New England Biolabs, USA) was used for protein expression and purification. *E. coli* were grown in Luria-Bertani (LB) medium (BD Biosciences, USA) supplemented with 100 *μg*/mL ampicillin (Sigma-Aldrich, USA).

### Preparation of Leptospiral Whole Cell Lysate

The *L. interrogans* serovars Lai, Canicola, Copenhageni, and the non-pathogenic serovar *L. biflexa* serovar Patoc were grown in liquid EMJH medium and harvested by centrifugation at 18,514 *g* for 10 mins. Cells were washed twice with 1X PBS pH 7.4 and pellets were resuspended in 5 mL/gram of BugBuster^®^ Protein Extraction Reagent (Sigma-Aldrich, USA) containing “Protease Inhibitor Cocktail with EDTA” (Roche, USA). Cell lysates were incubated on a rotating mixer for 15 minutes at room temperature. Insoluble cell debris was removed by centrifugation at 18,514 *g* for 20 minutes at 4°C. Supernatant were stored at -20°C until analysis.

### Computational Biology

N-and C-terminal amino acid sequences (LA3490, LA0620, LA1402, LA1400, and LA0591) of the PF07598 family were aligned using MAFFT (Multiple Alignment using Fast Fourier Transform) with using L-INS-i (accuracy-oriented) and visualized in Jalview v2.11.5 (https://www.jalview.org). The originally deposited LA1400 sequence was found to be incomplete in that it lacked sequence encoding the first 54 amino acids of the complete encoded protein. This conclusion was based on the use of clustal analysis to compare the amino acid sequences of *L. interrogans* serovar Lai LA1400 to LIC12340, the LA1400 ortholog in *L. interrogans* serovar Copenhageni strain FioCruz L1-130 (**Supplementary Figure 2**). The recombinant protein referred to in the present work as LA1400 is comprised of amino acids 31 to 54 derived from LIC12340, then LA1400-derived amino acids from position 55 to the end.

### Animals and Ethics Statement

All the animal experiments performed in this study were approved by the institutional Animal Care and Use Committee at Yale University (Protocol 2022-20243). All animal experiments were performed under Animal Biosafety Level (ABSL-2) conditions. All animals were under the supervision of an attending veterinarian and procedures were used to reduce pain and distress.

Three-week-old, specific pathogen-free, female C3H/HeJ mice were purchased from the Jackson Laboratories (ME, USA) and were maintained in a specific-pathogen-free environment at Yale Animal Resources Center. The mice were housed in individually ventilated microisolator cages with sterile, absorbent beddings changed twice weekly. The animals were fed and watered throughout the course of the experiment. Following *L. interrogans* serovar Canicola challenge, mice were weighed and monitored twice daily until the final endpoint. They were observed for loss of appetite, severe lassitude, difficulty in breathing, prostration, ruffled fur, and weight loss of 10%. Mice with these manifestations were euthanized by CO_2_ according to AAALAC/AVMA-approved procedures and considered to have met the endpoint of severe/lethal leptospirosis.

### Plasmid Constructs and Cloning

Synthetic *E. coli* codon-optimized genes were constructed by Gene Universal (https://www.geneuniversal.com) consisting of either the complete PF07598 genes encoding NCBI locus tag LA3490 (Uniprot: Q8F0K3), LA0620 (Q8F8D7), and LA1402 (Q8F6A7) from serovar Lai, and locus tag LIC12340 (Q72PX7) (Lai ortholog: LA1400), and LIC12985 (Q72N53) (Lai ortholog: LA0591) from serovar Copenhageni. Coding sequence, minus the predicted signal peptide or truncated 3490, an N-terminal domain, was synthesized and cloned into pET32b (+) (Gene Universal Inc., USA). LA3490, LA0620, LA1402, and t3490 were linked to mCherry (AST15061.1) *via* a glycine-serine hinge (Gly_4_Ser)_3_ and cloned into pET32b (+) (Gene Universal Inc., USA) between enterokinase cleavage sites for convenient removal of the mCherry fluorescent tag. Full-length LA1400 and LA0591 constructs were made without the mCherry fusion (**Figure 1B, C**). Prior to use, the sequence and the orientation of the genes in the constructs were verified by restriction digestion and sequencing.

### Expression and Purification of Recombinant Soluble PF07598 Antigens

Recombinant PF07598 protein constructs were expressed in SHuffle^®^T7 competent *E. coli* cells (New England Biolabs, USA). Transformants were sub-cultured into Luria-Bertani (LB) medium containing 100 *µ*g/mL ampicillin. Expression of PF07598 proteins were induced at OD of 0.6 *via* addition of 1 mM isopropyl-*β*-D-thiogalactoside (IPTG; Sigma-Aldrich, USA) and allowed to incubate at 16°C and 250 rpm for 24 h. Upon induction, cells were harvested and pellets were lysed in CelLytic^™^ B (Cell Lysis Reagent; Sigma-Aldrich, USA) containing 50 units benzonase nuclease (Sigma-Aldrich, USA), 0.2 *µ*g/mL lysozyme, non-EDTA protease inhibitor cocktail (Roche, USA) plus100 mM PMSF (Sigma-Aldrich, USA) for 30 minutes at 37°C. Supernatants and pellets were separated and then analyzed by 4-12% bis-tris sodium dodecyl sulfate-polyacrylamide gel electrophoresis (SDS-PAGE). Protein concentrations were determined by BCA assay (Bio-Rad, USA).

Recombinant PF07598 fusion and without fusion proteins were purified using a 5 mL pre-packed Ni-Sepharose AKTA Hi-TRAP column (GE Healthcare, USA) pre-equilibrated with a buffer containing 100 mM NaH_2_PO_4_, 10 mM Tris-HCl, 25 mM imidazole, and pH 8.0. PF07598 proteins bound to Hi-TRAP column were then eluted in the presence of 500 mM imidazole, and pH 8.0. Eluates were pooled, concentrated *via* a 10 kDa Amicon^®^ Ultra centrifugal filter, and further dialyzed overnight against 1X PBS (pH 7.4) with gentle stirring (350 rpm) at 4°C (10 kDa cutoff, Slide-A-Lyzer, Thermo Scientific™, USA). Purified recombinant PF07598 proteins were resolved in SDS-PAGE, verified by immunoblotting with mouse anti-His monoclonal-ALP conjugate (1:2,000 dilution; Santa Cruz Biotechnology, USA). Aliquot for boosters and SDS-PAGE were prepared from the single preparation and stored at −80°C to prevent repeated freeze-thawing.

### Animal Immunization, *Leptospira* Challenge and Sample Collection

C3H/HeJ mice were immunized *via* intramuscular (IM) route with recombinant PF07598 proteins (Viriyakosol et al., 2006). GLA–squalene–oil-in-water emulsion adjuvants (0.25 mg/mL) were procured from Infectious Disease Research Institute (IDRI), Seattle, WA, USA (http://www.idri.org). Immediately before injections, adjuvant was added to the recombinant protein or PBS to a final volume of 100 *μ*L and mixed by brief vortexing (Patra et al., 2015).

Mice were divided into four groups; G-I served as negative control and was injected with 1X phosphate buffer saline (PBS) mixed with adjuvant (EM082; 5 *µ*g GLA–squalene– oil-in-water emulsion). Similarly, G-II (t3490), G-III [VM mix, (LA3490, LA0620, LA1400, LA1402 and LA0591] and G-IV [VM unlabeled, (LA1400 and LA0591] were immunized with 25 *μ*g total antigen in equimolar ratio along with adjuvant (5 *µ*g GLA–squalene–oil-in-water emulsion) followed by two injections of 25 *μ*g of total antigen at 3-week intervals (**Figure 2**). Immunized mice were bled two weeks after the final immunization and to smooth out individual differences with groups, serum samples were pooled and measured for anti-VM antibodies in a serum known as pre-challenged bleed. All the groups were experimentally infected by intraperitoneal (IP) injection with 1x10^5^ organisms of a virulent, low passage isolate of *L. interrogans* serovar Canicola, strain LOCaS46, kindly provided by Dr. Alejandro de la Peña Moctezuma. Mice that survived infection were euthanized 13 days after infectious challenge. Blood was collected by terminal cardiac puncture and serum was isolated from whole blood. Serum was allowed to clot at room temperature and stored overnight at 4°C. Samples were then centrifuged at 11, 292 *g* for 15 minutes at 4°C. Serum was collected and stored at -80°C. Organs were collected and stored in RNALater at 4°C. Kidney and liver tissues were used for quantification of *L. interrogans* by quantitative PCR (qPCR).

### Evaluation of PF07598 Proteins-Induced Immunity by ELISA

Serum antibody responses to recombinant PF07598 proteins in immunized groups were quantified by ELISA (Chaurasia et al., 2018). Briefly, PF07598 antigens (LA3490, LA0620, LA1402, LA1400, and LA0598, respectively) in 100 *μ*L of bicarbonate/carbonate coating buffer were coated (250 ng) in 96-well microtiter ELISA plate (Corning, USA) and incubated at 4°C for overnight. Each set of antigens were incubated with pre–and post–immunized serum group (Group I–IV, 1:1000) for 1 h followed by goat anti-mouse IgG (Fc specific)–alkaline phosphatase conjugate (1:5000; KPL, USA) for 1 h, washed thrice with TBST and developed with p-Nitrophenyl phosphate (1-Step^™^ PNPP Substrate Solution; KPL, USA). The reaction was stopped with 2 M NaOH, and absorbance was read at 405 nm using a SpectraMax^®^ M2e Microplate Reader (Molecular Devices, USA). For whole cell ELISA, plate was coated with 500 ng/well cell free lysates. The controls included pre-bleed, pre-immunized serum samples, and antigen and antibody blanks.

### Sodium Dodecyl Sulfate-Polyacrylamide Gel Electrophoresis *(SDS-PAGE) and* Western Immunoblot Analysis

SDS-PAGE was done according to the method of Laemmli (1970). Immunoblot analysis was performed to determine whether sera from immunized animals recognize recombinant or native leptospiral PF07598 proteins. Purified recombinant PF07598 proteins or leptospiral whole cell lysate (120 mM NaCl induced and without induced) were transferred to nitrocellulose membranes and blocked for 2 h with 5% nonfat dry milk dissolved in 1X TBST buffer (AmericanBio, USA). The membrane was incubated with pooled sera from immunized groups (Group I–IV, 1:100) and controlled as pre-bleed and pre-immunize bleed for overnight at 4°C on rocker. They were probed with goat anti-mouse IgG (Fc specific)–alkaline phosphatase conjugate (1:5,000; KPL, USA) for 2.5 h, washed thrice with TBST, and developed with p-Nitrophenyl phosphate (1-Step^™^ PNPP Substrate Solution; KPL, USA). Monoclonal LipL32 antibody served as loading control (1:10,000 dilution).

### Quantitative PCR

DNA was extracted by dicing 40 ± 50 mg of kidney and liver tissues and suspending in 500 *μ*L of 1X PBS and all work was done under positive pressure in a location separate from the handling of *Leptospira* and PCR products in order to reduce risk of cross-contamination. Following tissue homogenization, total genomic DNA was extracted from the equivalent of 25 mg tissue using the DNeasy Blood and Tissue Kit (Qiagen, USA) per manufacturer’s instructions and eluted in 50 *μ*L of elution buffer. *L. interrogans s*erovar Canicola at a density of 2x10^7^ leptospires/mL grown in 5 mL of EMJH culture medium. Cells were harvested and DNA was extracted for standard curves using the same DNeasy Blood and Tissue Kit (Qiagen, USA).

The concentration of eluted DNA was determined using a NanoDrop Spectrophotometer ND-1000 (NanoDrop Technologies, USA). All DNA samples were kept at -80°C until use. Serial dilution (1x10° to 1x10^7^ genomic equivalents (GEq)/5 *μ*L) of DNA was prepared and *L. interrogans* Serovar Canicola genome was quantified by qPCR using 2X iQ5 SYBR Green supermix (Bio-Rad, CA, USA) with 5 pmol forward (5’-TCT GTG ATC AAC TAT. TAC GGA TAC-3’) and reverse (5’-ATC CAA GTA TCA AAC CAA TGT GG -3’) LipL32 primer. Four microliters of standard or sample DNA was added to 10 *μ*L PCR mix and the reaction was subjected to amplification in the CFX96 Real-time PCR Detection System (Bio-Rad, USA) using the following program: 3 min at 95°C, 0.10 min at 95°C, 0.30 min at 62°C, followed by 44 cycles at 1.00 min at 72°C then final extension 7 min at 72°C. A standard curve was generated using Bio-Rad iQCycler5 software and the number of GEq was extrapolated from the threshold cycle (CT) values. A negative result was assigned where no amplification occurred or if the CT value was greater than 3 SD+Ct. Data are presented as the number of *L. interrogans* GEq per gram of tissue.

### Statistical Analysis

All experiments were performed in triplicate and repeated twice. The Kruskal-Wallis test was used to determine significant differences in the number of bacteria in the kidney or liver among the survivors from different immunization groups. The results were analyzed by the non-parametric Mann–Whitney test to determine significant differences between individual groups and were considered as statistically significant when *p < 0.05, p < 0.001 p < 0.0001*. All analyses and graphs were generated using Graph Prism version 8 (GraphPad Software, Inc., USA).

## Data Availability Statement

The original contributions presented in the study are included in the article/**Supplementary Material**. Further inquiries can be directed to the corresponding author.

## Ethics Statement

The animal study was reviewed and approved by Yale Institutional Animal Care and Use Committee.

## Author Contributions

Conceptualization: RC and JV. Data curation: RC, XG, and JV. Formal analysis: RC, XG, GD, and JV. Funding acquisition: JV. Investigation: RC, AS, XG, and JV. Methodology: RC, AS, XG, and JV, Resources: GD and JV. Supervision: GD and JV. Visualization: RC, AS, XG, and JV. Writing - original draft: RC. Writing - review & editing: RC, AS, XG, GD, and JV. All authors contributed to the article and approved the submitted version.

## Funding

This work was supported by the United States Public Health Service through National Institutes of Health, NIAID grants R01AI108276 and U19AI115658.

## Conflict of Interest

The work reported here has been filed in patent applications from Yale University. JV and spouse have an equity interest in LeptoX BioPharma, Inc. which may have a future interest in licensing this work.

The remaining authors declare that the research was conducted in the absence of any commercial or financial relationships that could be construed as a potential conflict of interest.

## Publisher’s Note

All claims expressed in this article are solely those of the authors and do not necessarily represent those of their affiliated organizations, or those of the publisher, the editors and the reviewers. Any product that may be evaluated in this article, or claim that may be made by its manufacturer, is not guaranteed or endorsed by the publisher.
