# Supplementary material for: Vaccination With Leptospira interrogans PF07598 Gene Family-Encoded Virulence Modifying Proteins Protects Mice From Severe Leptospirosis and Reduces Bacterial Load in the Liver and Kidney
Source: Front Cell Infect Microbiol. 2022 Jun 28;12:926994. doi: 10.3389/fcimb.2022.926994 (PMC9274288; doi:10.3389/fcimb.2022.926994)
Supplement: Supplementary file 1 [file DataSheet_1.pdf]

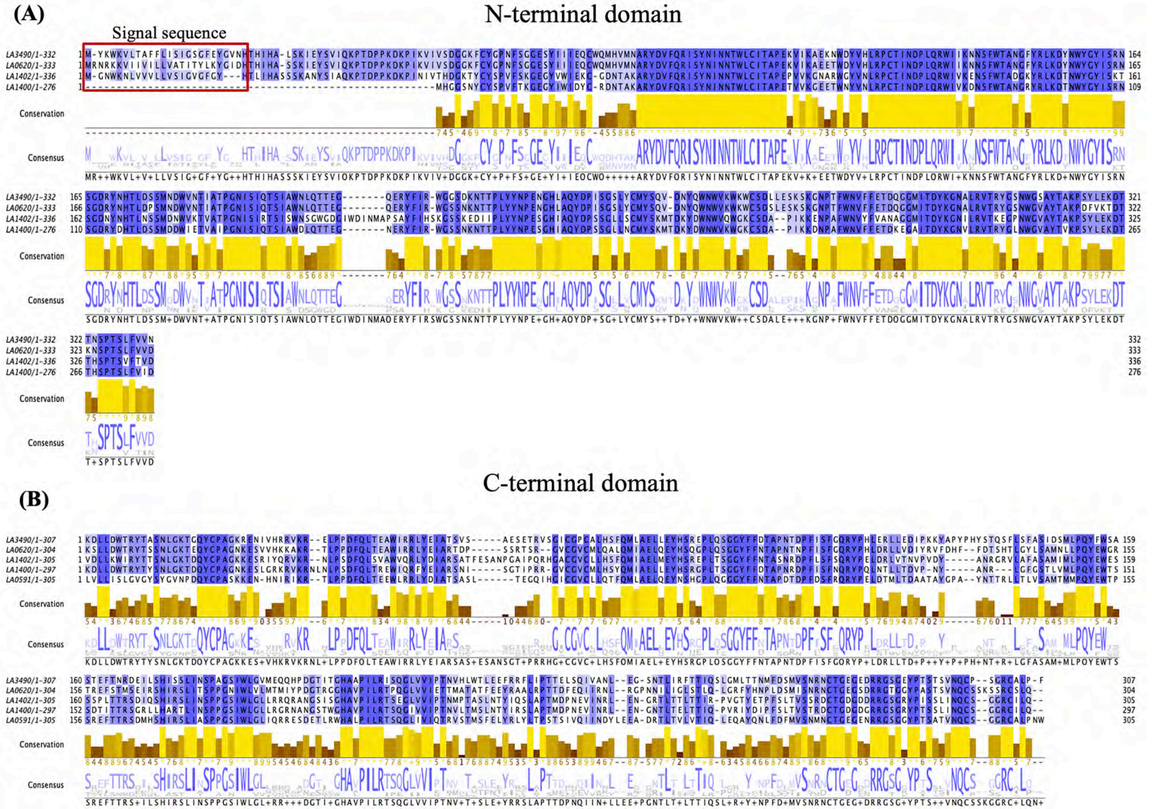
(A)
N-terminal domain
Signal sequence
LA3490/1-332
LA0620/1-333
LA1402/1-336
LA1400/1-276
Conservation
Consensus
C-terminal domain
(B)
LA3490/1-307
LA0620/1-304
LA1402/1-305
LA1400/1-297
LA0591/1-305
Conservation
Consensus

Comparison of the VM protein ortholog, LA1400 (UniProt Q8F6A9), from *Leptospira interrogans* serovar Lai, with its ortholog, LIC12340(Q72PX7), from Leptospira interrogans serogroup Icterohaemorrhagiae serovar copenhageni (strain Fiocruz L1-130.

From UniProt

>tr|Q8F6A9|Q8F6A9_LEPIN Ricin B-type lectin domain-containing protein OS=Leptospira interrogans serogroup Icterohaemorrhagiae serovar Lai (strain 56601) OX=189518 GN=LA_1400 PE=4 SV=2
MHGGSNYCYSPVFTKGEGYIWIDYCRDNTAKARYDVFQRISYNINNTWLCITAPETVVKG
EETWNYVNLRPCTINDPLQRWIVKDNSFWTANGRYRLKDTNWYGYISRNSGDRYDHTLDS
SMDDWIETVAIPGNISIQTSIAWDLQTTEGNERYFIRWGSSNKNTTPLYYNPESGHIAQY
DPSSGLLNCMYSKMTDKYDWNWVKWGKCSDAPIKKDNPAFWNVFFETDKEGAITDYKGNV
LRVTRYGLNWGVAYTVKPSYLEKDTTHSPTSLFVIDKDLLDWTRYTYSNLGKTDQYCPAG
NKESLGRKRVKRNLNLPSDFQLTREWIQRFYEIARSNISGTIPRRGVCGVCMLHSYQMIA
ELLEYHSRGPLTSGGYFFDTAPNRDPFISFNQRYPQLNTLLTDVPNYANRLGFGSTLVML
PQYEWTSSDTITTRSGRLLHARTLINSPPGSIWLGLLRGRNANGSTWGHAVPILRTSQGI
VVIPTNVLTMSLNTYIRSLAPTMDPNEVINRLENGNTLTELTTIQPVRIYDIPFSLTVST
RDCTGDGDGRRGSGRYPTSSLINQCSGGRCILQ

>>tr|Q72PX7|Q72PX7_LEPIC Uncharacterized protein OS=Leptospira interrogans serogroup Icterohaemorrhagiae serovar copenhageni (strain Fiocruz L1-130) GN=LIC_12340 PE=4 SV=1
MGRWIVLRVSLLVLIGIGFEYGINHTSINA^SSKSDYSIAQKPADQPKDKSIQVVMHGGSNYCYSPVFTKGEGYIWI
DYCSDNTAKARYDVFQRISYNINNTWLCITAPETVVKGEETWNYVNLRPCTINDPLQRWIVKDNSFWTANGRYRLKD
TNWYGYISRNSGDRYDHTLNSSMDDWIKTVAIPGNISIQTSIAWDLQTTEGNERYFIRWGSSNKNTTPLYYNPESGH
IAQYDPSSGLLNCMYSKMTDKYDWNWVKWGKCSDAPIKKDNPAFWNVFFETDKEGAITDYKGNVLRVTRYGLNWGVA
YTVKPSYLEKDTTHSPTSLFVIDKDLLDWTRYTYSNLGKTDQYCPAGNKESLVRKRVKRNLNLPSDFQLTREWIQRL
YEIARSSISRAIPCRGVCGVCMLHSYQMIAELLEYHSRGPLTGGGYFFDTAPNRDPFISFNQRYPQLNALLTNVPSY
ANRPGFGSTLVMLPQYEWTSSDTITTRSGRLLHARSLINSPPGSIWLGLLRGRDADGSTWGHAVPILRTSQGIVVIP
TNSPTMSLNTYIRSLAPTMDPNEVINRLENGSTLTELTTIQPVRIYDIPFSLTVSTRDCTGDGDGRRGSGRYPTSSL
INQCSGGRCILQ

CLUSTALW Alignment of LA1400 and LIC12340
Predicted signal peptide in bold, not included in recombinant protein construct. The recombinant “LA1400” used in the present study starts with SSKSDYS and ends with RCILQ.

```
LA_1400         ------------------------------------------------------MHGGSN
LIC_12340       MGRWIVLRVSLLVLIGIGFEYGINHTSINASSKSDYSIAQKPADQPKDKSIQVVMHGGSN
                                                                      ******

LA_1400         YCYSPVFTKGEGYIWIDYCRDNTAKARYDVFQRISYNINNTWLCITAPETVVKGEETWNY
LIC_12340       YCYSPVFTKGEGYIWIDYCSDNTAKARYDVFQRISYNINNTWLCITAPETVVKGEETWNY
                ******************* ****************************************

LA_1400         VNLRPCTINDPLQRWIVKDNSFWTANGRYRLKDTNWYGYISRNSGDRYDHTLDSSMDDWI
LIC_12340       VNLRPCTINDPLQRWIVKDNSFWTANGRYRLKDTNWYGYISRNSGDRYDHTLNSSMDDWI
                ****************************************************:*******

LA_1400         ETVAIPGNISIQTSIAWDLQTTEGNERYFIRWGSSNKNTTPLYYNPESGHIAQYDPSSGL
LIC_12340       KTVAIPGNISIQTSIAWDLQTTEGNERYFIRWGSSNKNTTPLYYNPESGHIAQYDPSSGL
                :***********************************************************

LA_1400         LNCMYSKMTDKYDWNWVKWGKCSDAPIKKDNPAFWNVFFETDKEGAITDYKGNVLRVTRY
LIC_12340       LNCMYSKMTDKYDWNWVKWGKCSDAPIKKDNPAFWNVFFETDKEGAITDYKGNVLRVTRY
```

```
                    ************************************************************

LA_1400             GLNWGVAYTVKPSYLEKDTTHSPTSLFVIDKDLLDWTRYTYSNLGKTDQYCPAGNKESLG
LIC_12340           GLNWGVAYTVKPSYLEKDTTHSPTSLFVIDKDLLDWTRYTYSNLGKTDQYCPAGNKESLV
                    *********************************************************** 

LA_1400             RKRVKRNLNLPSDFQLTREWIQRFYEIARSNISGTIPRRGVCGVCMLHSYQMIAELLEYH
LIC_12340           RKRVKRNLNLPSDFQLTREWIQRLYEIARSSISRAIPCRGVCGVCMLHSYQMIAELLEYH
                    ***********************:******.** :** **********************

LA_1400             SRGPLTSGGYFFDTAPNRDPFISFNQRYPQLNTLLTDVPNYANRLGFGSTLVMLPQYEWT
LIC_12340           SRGPLTGGGYFFDTAPNRDPFISFNQRYPQLNALLTNVPSYANRPGFGSTLVMLPQYEWT
                    ******.*************************:***:**.**** ***************

LA_1400             SSDTITTRSGRLLHARTLINSPPGSIWLGLLRGRNANGSTWGHAVPILRTSQGIVVIPTN
LIC_12340           SSDTITTRSGRLLHARSLINSPPGSIWLGLLRGRDADGSTWGHAVPILRTSQGIVVIPTN
                    ****************:*****************:*:***********************

LA_1400             VLTMSLNTYIRSLAPTMDPNEVINRLENGNTLTELTTIQPVRIYDIPFSLTVSTRDCTGD
LIC_12340           SPTMSLNTYIRSLAPTMDPNEVINRLENGSTLTELTTIQPVRIYDIPFSLTVSTRDCTGD
                      ***************************.******************************

LA_1400             GDGRRGSGRYPTSSLINQCSGGRCILQ
LIC_12340           GDGRRGSGRYPTSSLINQCSGGRCILQ
                    ***************************
```
